# Identification of the Immunodominant Regions of *Staphylococcus aureus* Fibronectin-Binding Protein A

**DOI:** 10.1371/journal.pone.0095338

**Published:** 2014-04-15

**Authors:** Qian-Fei Zuo, Chang-Zhi Cai, Hong-Lei Ding, Yi Wu, Liu-Yang Yang, Qiang Feng, Hui-Jie Yang, Zhen-Bo Wei, Hao Zeng, Quan-Ming Zou

**Affiliations:** 1 National Engineering Research Center of Immunological Products, Department of Microbiology and Biochemical Pharmacy, College of Pharmacy, Third Military Medical University, Chongqing, PR China; 2 Department of Biological Engineering and Chemical Engineering, Chongqing University of Education, Chongqing, PR China; National Institutes of Health, United States of America

## Abstract

*Staphylococcus aureus* is an opportunistic bacterial pathogen responsible for a diverse spectrum of human diseases and a leading cause of nosocomial and community-acquired infections. Development of a vaccine against this pathogen is an important goal. The fibronectin binding protein A (FnBPA) of *S. aureus* is one of multifunctional ‘microbial surface components recognizing adhesive matrix molecules' (MSCRAMMs). It is one of the most important adhesin molecules involved in the initial adhesion steps of *S. aureus* infection. It has been studied as potential vaccine candidates. However, FnBPA is a high-molecular-weight protein of 106 kDa and difficulties in achieving its high-level expression in vitro limit its vaccine application in *S. aureus* infection diseases control. Therefore, mapping the immunodominant regions of FnBPA is important for developing polyvalent subunit fusion vaccines against *S. aureus* infections. In the present study, we cloned and expressed the N-terminal and C-terminal of FnBPA. We evaluated the immunogenicity of the two sections of FnBPA and the protective efficacy of the two truncated fragments vaccines in a murine model of systemic *S. aureus* infection. The results showed recombinant truncated fragment F1_30-500_ had a strong immunogenicity property and survival rates significantly increased in the group of mice immunized with F1_30-500_ than the control group. We futher identified the immunodominant regions of FnBPA. The mouse antisera reactions suggest that the region covering residues 110 to 263 (F1B_110-263_) is highly immunogenic and is the immunodominant regions of FnBPA. Moreover, vaccination with F1B_110-263_ can generate partial protection against lethal challenge with two different *S. aureus* strains and reduced bacterial burdens against non-lethal challenge as well as that immunization with F1_30-500_. This information will be important for further developing anti- *S. aureus* polyvalent subunit fusion vaccines.

## Introduction

*Staphylococcus aureus* is an opportunistic bacterial pathogen responsible for a diverse spectrum of human diseases [1], [2], which are from mild culture-confirmed skin and soft tissue infections to life-threatening and highly invasive disease [3], [4], [5]. Multidrug-resistant *S. aureus* infections are ever increasing [6]. Not only has *S. aureus* resistance to methicillin become more common, but numerous isolates with reduced susceptibility to vancomycin have been reported [7], [8]. Because *S. aureus* cannot always be controlled by antibiotics and MRSA isolates are becoming increasingly prevalent in the community [9], [10], hence immunotherapeutic strategies, such as a vaccine, are sorely needed.

*S. aureus* possesses over 50 virulence factors [11], enabling the bacterium to adapt to a variety of host niches and to cause a multitude of diverse infections. These factors include a number of ‘microbial surface components recognizing adhesive matrix molecules' (MSCRAMMs), capsular polysaccharides (CPs) and staphylococcal toxins [12], [13], [14]. MSCRAMMs are anchored to bacterial cell wall peptidoglycan by a mechanism that involves the enzyme sortase and a sorting signal that comprises a conserved LPXTG motif. They recognize and bind to human extracellular matrix components such as fibrinogen or fibronectin. A number of MSCRAMMs, for example, Iron-responsive surface determinant A & H [13], Iron-responsive surface determinant B [15], Serine aspartate repeat protein D & E [16], Collagen adhesion [17], Clumping factor A [18], [19], Clumping factor B [20], have been tested in *in vivo* animal models and generate partial protection immune responses against *S. aureus* challenge.

The fibronectin binding protein A (FnBPA) of *S. aureus* is one of multifunctional MSCRAMMs which recognize fibronectin, fibrinogen and elastin. The protein contains an N-terminal region that binds fibrinogen and elastin [21], [22], and a C-terminal domain that interacts with fibronectin [23]. It is one of the most important adhesin molecules involved in the initial adhesion steps of *S. aureus* infection [24]. Therefore, it has been studied as potential vaccine candidates. Immunizations of rats with a truncated D2-domain of the fibrinonectin binding protein induced protection against endocarditis [25]. Mice that were immunized with a combination of collagen adhesin and fibrinonectin binding protein survived significantly longer following a challenge with *S. aureus* than nonimmunized mice [26]. However, FnBPA is a high-molecular-weight protein of 106 kDa and difficulties in achieving its high-level expression in vitro limit its vaccine application in *S. aureus* infection diseases control. Particularly, the expression of multiple protein fusion vaccine which contains FnBPA becomes unrealistic. Therefore, mapping the immunodominant regions of FnBPA is important for developing polyvalent subunit fusion vaccines against *S. aureus* infections.

In the present study, N-terminal and C-terminal of FnBPA (F1_30-500_ and F2_501-941_) were cloned and expressed. We evaluated the immunogenicity of the two sections of FnBPA by an enzyme-linked immunosorbent assay (ELISA) and the protective efficacy of the two truncated fragments vaccines in a murine model of systemic *S. aureus* infection. Moreover, we mapped the immunodominant regions of the two truncated fragments, and we compared the protective efficacy of the immunodominant region of the FnBPA with the truncated fragment (F1_30-500_). This information will be important for further developing anti- *S. aureus* polyvalent subunit fusion vaccines.

## Materials and Methods

### Ethics Statement

All of the animal experiments were approved by the Animal Ethical and Experimental Committee of the Third Military Medical University (chongqing; permit number 2011-04). All surgery was performed under sodium pentobarbital anesthesia, and animals were sacrificed at the time points indicated below using CO_2_ inhalation. All efforts were made to minimize suffering.

### Bacterial strains and culture conditions

*S. aureus* strain MRSA252 was obtained from the American Type Culture Collection (Manassas, VA, USA). MRSA strain WHO-2 (WHO-2) was kindly provided by Professor Hong Zou, The Third Military Medical University (chongqing, China). They were used for the murine systemic infection model. The bacteria were grown in tryptic soy broth at 37°C for 6 h, centrifuged at 5000 g for 5 min, and subsequently washed with sterile phosphate-buffered saline (PBS). The washed bacteria were diluted with PBS to an appropriate cell concentration as determined by spectrophotometry at 600 nm.

### Cloning and expression of recombinant fragments

Genomic DNA was isolated from *S. aureus* strain MRSA252 and used as the PCR template. All the fragments (F1_30-500_, F1A_30-173_, F1B_110-263_, F1C_195-333_, F1D_264-372_, F1E_373-500_, F2_501-941_, F2A_501-616_, F2B_586-756_, F2C_663-865_, F2D_738-900_, and F2E_805-941_) genes were amplified by PCR using the primers listed in Table 1. For all of the amplified genes, BamHI and NotI sites were incoporated at the beginning and end of the PCR products by primers. Double digested PCR products were ligated into pGEX-6P-2 vector and transformed with the *Escherichia coli* Xl/blue strain. The resulting constructs were transformed into *Escherichia coli* strain BL21(DE3) for isopropyl-β-D-1-thiogalactopyranoside (IPTG)-induced expression and were expressed in fusion with glutathione-S-transferase (GST). The fusion proteins were extracted by lysing the bacteria via sonication in a Triton-X100 lysis buffer (1%TritonX-100, 75 units/ml of Aprotinin, 1.6 mM Pepstatin, 20 mM Leupeptin and 1 mM PMSF) as described previously [27]. After a high-speed centrifugation to remove debris, the fusion protein-containing supernatants were either directly added to glutathione-coated microplates for measuring their reactivity with mouse sera in an ELISA as described below or further purified using glutathione-conjugated agarose beads (Pharmacia).

**Table 1:** A list of oligonucleotide primers used in the construction of plasmids expressing recombinant fibronectin-binding protein A fragments.

Fragment	Directions	Sequence 5'-3'	Restriction site
F1	Forward	CGCGGATCCATGGGACAAGATAAAGAAGCTGCA	*Bam*HI
F1	Reverse	TTTTCCTTTTGCGGCCGCCTATCCATTATCCCATGTTAATGTAT	*Not*I
F1A	Forward	CGCGGATCCATGGGACAAGATAAAGAAGCTGCAG	*Bam*HI
F1A	Reverse	TTTTCCTTTTGCGGCCGCCTACACGTGGCTTACTTTCTAATGC	*Not*I
F1B	Forward	CGCGGATCCATGGTAGAAACAGTTAAAGAAGAGGTAGTTA	*Bam*HI
F1B	Reverse	TTTTCCTTTTGCGGCCGCCTATGGAACTTTTCTTGTAGTTGCTAC	*Not*I
F1C	Forward	CGCGGATCCATGACAGATGTGACAAGTAAAGTTACAGTG	*Bam*HI
F1C	Reverse	TTTTCCTTTTGCGGCCGCCTACATAGTTCCTGATGTTTCTTTGC	*Not*I
F1D	Forward	CGCGGATCCATGGATATTAAAAATGGATCATTAGTTATGG	*Bam*HI
F1D	Reverse	TTTTCCTTTTGCGGCCGCCTAATTTATAGGCTTAATATATGCTACGTG	*Not*I
F1E	Forward	CGCGGATCCATGGGAAACAATTCAGATAGTGTTACTGT	*Bam*HI
F1E	Reverse	TTTTCCTTTTGCGGCCGCCTATCCATTATCCCATGTTAATGTATAG	*Not*I
F2	Forward	CGCGGATCCATGTTAGTTTTATATAGTAATAAAGCTAA	*Bam*HI
F2	Reverse	TTTTCCTTTTGCGGCCGCCTAACCTTTGTTTGTTGATTCTTCTC	*Not*I
F2A	Forward	CGCGGATCCATGTTAGTTTTATATAGTAATAAAGCTAATGG	*Bam*HI
F2A	Reverse	TTTTCCTTTTGCGGCCGCCTATTCAAAGTCAATTGGATTTGATTC	*Not*I
F2B	Forward	CGCGGATCCATGGATATCGATTACCATACTGCTGTG	*Bam*HI
F2B	Reverse	TTTTCCTTTTGCGGCCGCCTAGAATGACTGATTACCGCTATTTTG	*Not*I
F2C	Forward	CGCGGATCCATGGGCGCAGTGAGCGACCATAC	*Bam*HI
F2C	Reverse	TTTTCCTTTTGCGGCCGCCTATGGCTCACTTGGCACTTCTG	*Not*I
F2D	Forward	CGCGGATCCATGGATATTAAGAGTGAATTAGGTTACGAAG	*Bam*HI
F2D	Reverse	TTTTCCTTTTGCGGCCGCCTAACCTTGTTCCACTGGTTTAGAAG	*Not*I
F2E	Forward	CGCGGATCCATGTATCAATTCGGTGGACACAACA	*Bam*HI
F2E	Reverse	TTTTCCTTTTGCGGCCGCCTAACCTTTGTTTGTTGATTCTTCTCC	*Not*I

### Purification of recombinant proteins and removal of endotoxin

GST-tagged proteins were affinity-purified from cleared lysates with glutathione-Sepharose. Then the recombinant proteins were purified by Capto™ MMC. The protein eluate was subjected to endotoxin removal by Triton X-114 phase separation.

### Immunization and challenge infection

**Figure 1:**
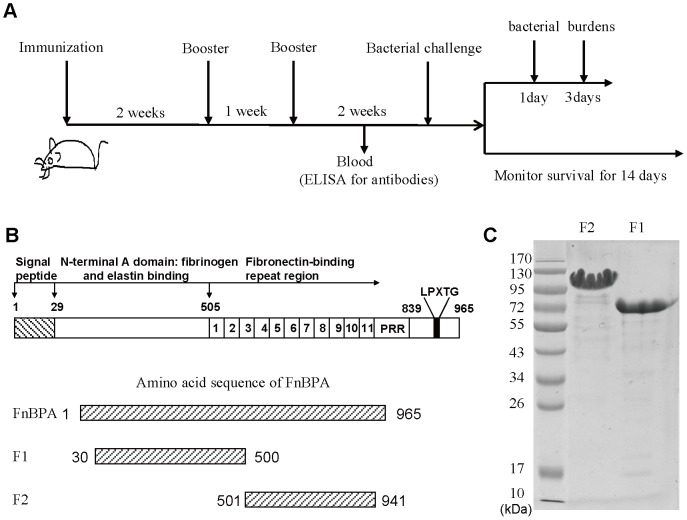
Immunization schedule and expression of recombinant truncated fragment proteins. (A) Diagrams showing the experimental design of the immunization schedule for the measurement of antibodies, the survival rates up to 14 days after bacterial challenge and the bacterial budens on days 1 and 3 after bacterial infection. (B) Structural organization of the fibronectin-binding protein, FnBPA from *S. aureus* strain MRSA252 and schematic diagram illustrating the primary structure of the FnBPA_1-965_, F1_30-500_(F1) and F2_501-941_(F2). (C) Recombinant GST-tagged F1 and F2 were purified by affinity chromatography and analyzed by SDS-PAGE.

BALB/c mice (6–8 weeks of age) were injected intramuscularly twice with 50 µl of the emulsion containing 20 µg of protein or with PBS plus adjuvant alum (Pierce) as a control on days 0, 14, and 21. To determine the survival rates after *S. aureus* infection, BALB/c mice were anesthetized with sodium pentobarbital before injection and were infected with *S. aureus* (1×10^9^ CFU per mouse) on day 35. The survival rates were monitored for 14 days after infection. The condition of the mice were monitored and recorded at 8, 16, and 24 o'clock every day. In the survival study, although the animals died as a direct result of the intervention, our research design included plans to consider humane euthanasia for mice that were observed to be suffering severe disease or became moribund during the 14 day survival study. In detail, all animals in the survival study were sacrificed by CO_2_ asphyxiation when they became moribund as defined by a combination of ruffled fur, hunched back and dulled response to stimuli, such as finger probing. At the completion of all experiments, survivors were sacrificed by CO_2_ overdose in accordance with IACUC policy. To determine the bacterial numbers, BALB/c mice were infected with 2.5×10^8^ of *S. aureus* strain MRSA252, and the target tissues were assessed for bacterial colonization at 1 and 3 days after infection (as shown in Figure 1A).

### ELISA for specific antibodies

F1_30-500_(F1) and F2_501-941_(F2)-specific antibodies were measured in sera obtained from mice by ELISA as described previously [15]. Purified F1 and F2 were used to coat the ELISA plates at a concentration of 10 µg/ml in phosphate buffer, pH 7.4. To detect the reactivities of antisera from the 20 mice with the 12 fusion proteins, a protein array ELISA was used, as described elsewhere [27], [28]. Briefly, bacterial lysates containing the GST fusion proteins were added directly to 96-well microplates precoated with glutathione (Pierce, Rockford, IL) to allow GST to interact with the glutathione. After washing to remove excess fusion proteins and blocking with 2.5% nonfat milk (in PBS), individual mouse serum samples were applied to the microplates after the appropriate dilutions. The serum antibody binding to antigens was detected with a goat anti-mouse IgG conjugated with horseradish peroxidase (HRP), in combination with the soluble substrate 2, 2'-azino-bis(3-ethylbenzothiazoline-6-sulforic acid) diammonium salt (ABTS) (Sigma), and quantitated by reading the absorbance at 450 nm using a microplate reader.

### Bacterial burden

On days 1 and 3 after infection, the kidneys were harvested for the determination of the bacterial burden. The bacterial numbers in the organs were enumerated by preparing organ homogenates in PBS and plating 10-fold serial dilutions on tryptic soy agar (BD Diagnosis System). The colonies were counted after 24 h of incubation at 37°C.

### Antibody analysis for opsonic killing activity

Rabbits immunized with *S. aureus* antigens were tested for functional activity in a classic *in vitro* opsonophagocytic killing assay. Briefly, HL-60 cells were cultured, washed, counted, examined for viability by trypan blue exclusion, and the final cell concentration adjusted to 1–2×10^6^ HL-60 cells per ml. Cross-reactive antibodies in infant rabbit serum were removed by incubation with suspensions of *S. aureus* MRSA252 by mixing at 4°C for 30 min. Serum was then centrifuged, filter-sterilized, and used as a source of complement. *S. aureus* MRSA252 was adjusted to 1–2×10^5^ CFU per ml. Equal volumes (100 µl) of HL-60 cells, complement, bacteria, and diluted antibodies were mixed and incubated at 37°C for 90 min prior to dilution, agar plating, and bacterial enumeration. Bacterial killing was calculated as the percent difference in CFU between samples without or with HL-60 cells.

### Statistical analysis

The non-parametric log rank test was utilized to determine differences in the survival times. The Mann-Whitney U test was used to compare bacterial burden. Analyses were performed using GraphPad Prism 5.0 (GraphPad Software). *P*<0.05 was considered significant.

## Results

### Cloning and expression of recombinant truncated fragment proteins F1_30-500_(F1) and F2_501-941_(F2)

As shown in Figure 1B, structural organization of the fibronectin-binding protein, FnBPA from *S. aureus* strain MRSA252 was given and the coordinates of FnBPA were defined based on the coordinates of the A domain and the Fn binding repeats domains of FnBPA from *S. aureus* strain 8325. The F1 and F2 genes were amplified by PCR. The recombinant gene fragments were cloned into the pGEX-6P-2 vector. Following IPTG induction, the recombinant fragments were expressed as soluble proteins. Recombinant GST-tagged F1 and F2 were purified by affinity chromatography and analyzed by SDS-PAGE (Figure 1C). The results suggest that the aim proteins expressed at high level in soluble form.

### Immunization with the recombinant truncated fragment proteins induced different antibody responses

**Figure 2:**
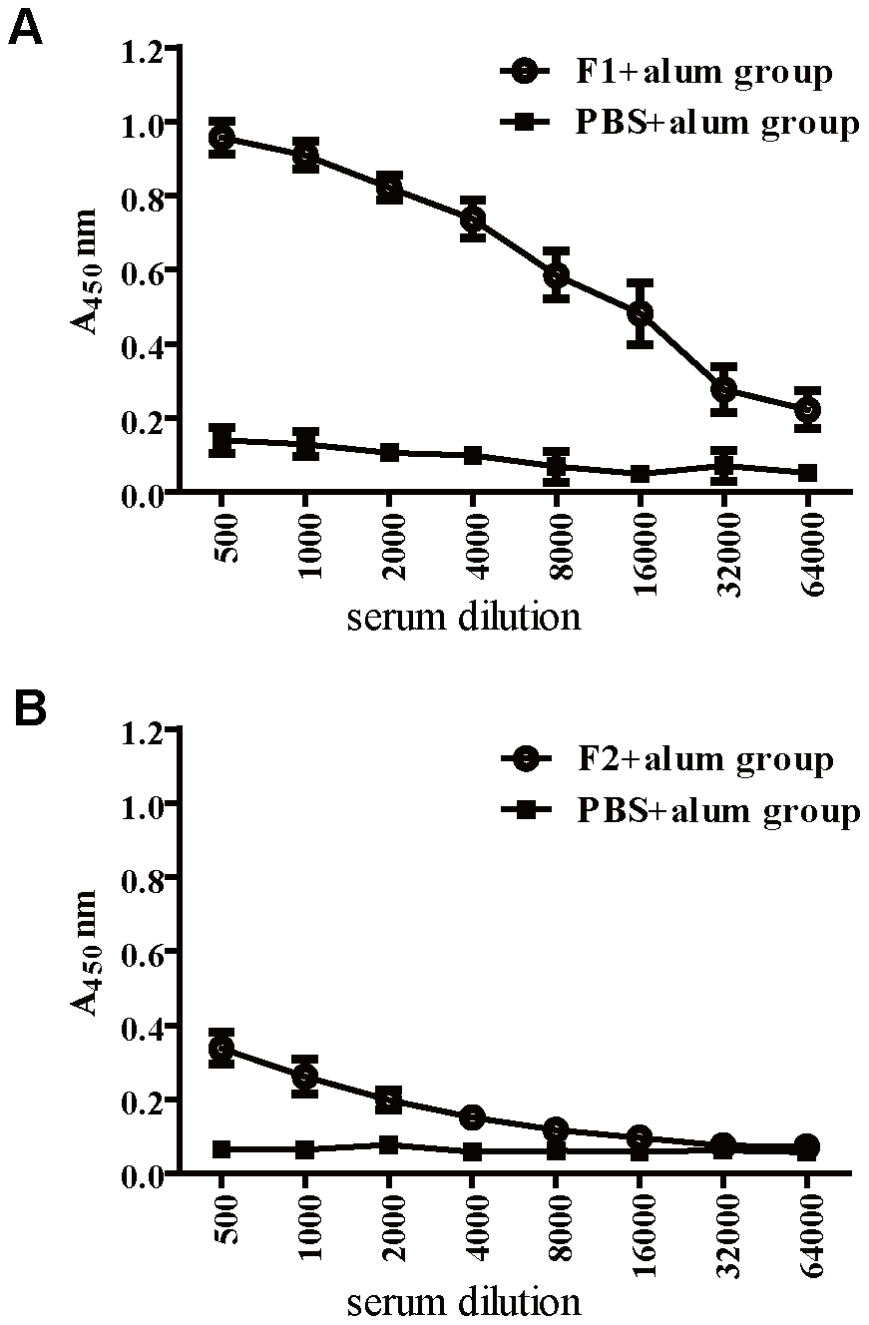
Production of anti-F1 and F2 antibody by BALB/c mice immunized with F1 or F2. The anti-serum was collected at the first week after the last immunization. Each group has six mice. (A) Elisa titration of antibodies directed against F1 in sera from mice immunised with F1 and alum or PBS and alum. The ELISA plates were coated with F1 as antigen. (B) Elisa titration of antibodies directed against F2 in sera from mice immunised with F2 and alum or PBS and alum. The ELISA plates were coated with F2 as antigen. Standard deviations are indicated by bars.

To evaluate the immunogenicity of the recombinant protein in actively immunized mice, the titration of specific antibodies against the different recombinant proteins were determined by ELISA one week after the last booster. Compared to alum group, immunization with the F1 induced a high level antibody response (Figure 2A). However, immunization with the F2 induced a low level humoral immune response to F2 (Figure 2B). The results indicated the recombinant truncated fragment F1 had a strong immunogenicity property and the recombinant truncated fragment F2 had a poor antigenic property.

### Immunization with the recombinant protein vaccine (F1) generated protective immunity against MRSA252 challenge

**Figure 3:**
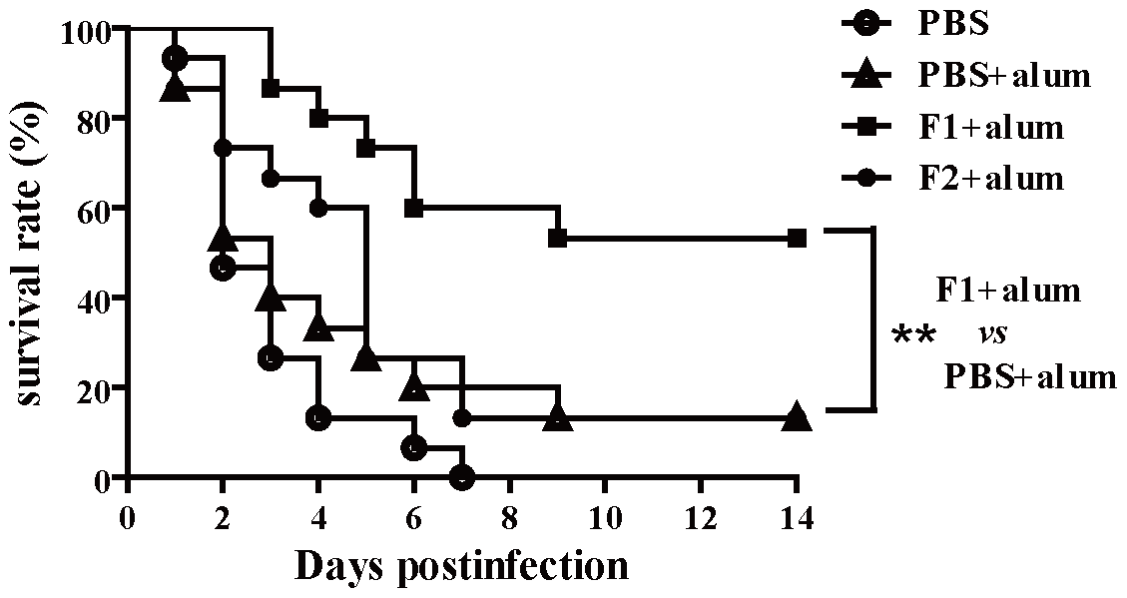
Immunization with the recombinant protein vaccine (F1) generated protective immunity against MRSA252 challenge. BALB/c mice (n = 15) were immunized with individual antigens (F1, F2) and alum adjuvant. The animals were challenged by intravenous injection of MRSA252 (1×10^9^ CFU) and were monitored for 14 days. Compared with animals receiving antigen-free PBS and the adjuvant alone, the significance of the protective immunity generated by the various antigens was measured with a log rank test: F1, *P* = 0.0038; F2, *P* = 0.5375. The asterisks represent a statistically significant difference (**P<0.01). Representative results from one of three independent experiments are shown.

he mice were immunized with F1 or F2 three times at one- to two-week intervals. Fourteen days after the last immunization, the mice were infected via the tail vein with 1×10^9^ cells of *S. aureus* MRSA252. The mice vaccinated with the F1 antigen displayed higher survival rates (53.3% at 14 days) than the alum adjuvant control group (13.3% survival). However, The mice vaccinated with the F2 antigen displayed similarly survival rates (13.3% at 14 days) compared to the alum adjuvant control group (13.3% survival) (Figure 3). The significance of protective immunity generated by the different antigens was measured with a log rank test (F1, *P* = 0.0038; F2, *P* = 0.5375.). These results suggest that immunization with a recombinant F1 vaccine can generate partial protection against lethal challenge with *S. aureus* MRSA252.

### Mapping the immunodominant regions of FnBPA

**Figure 4:**
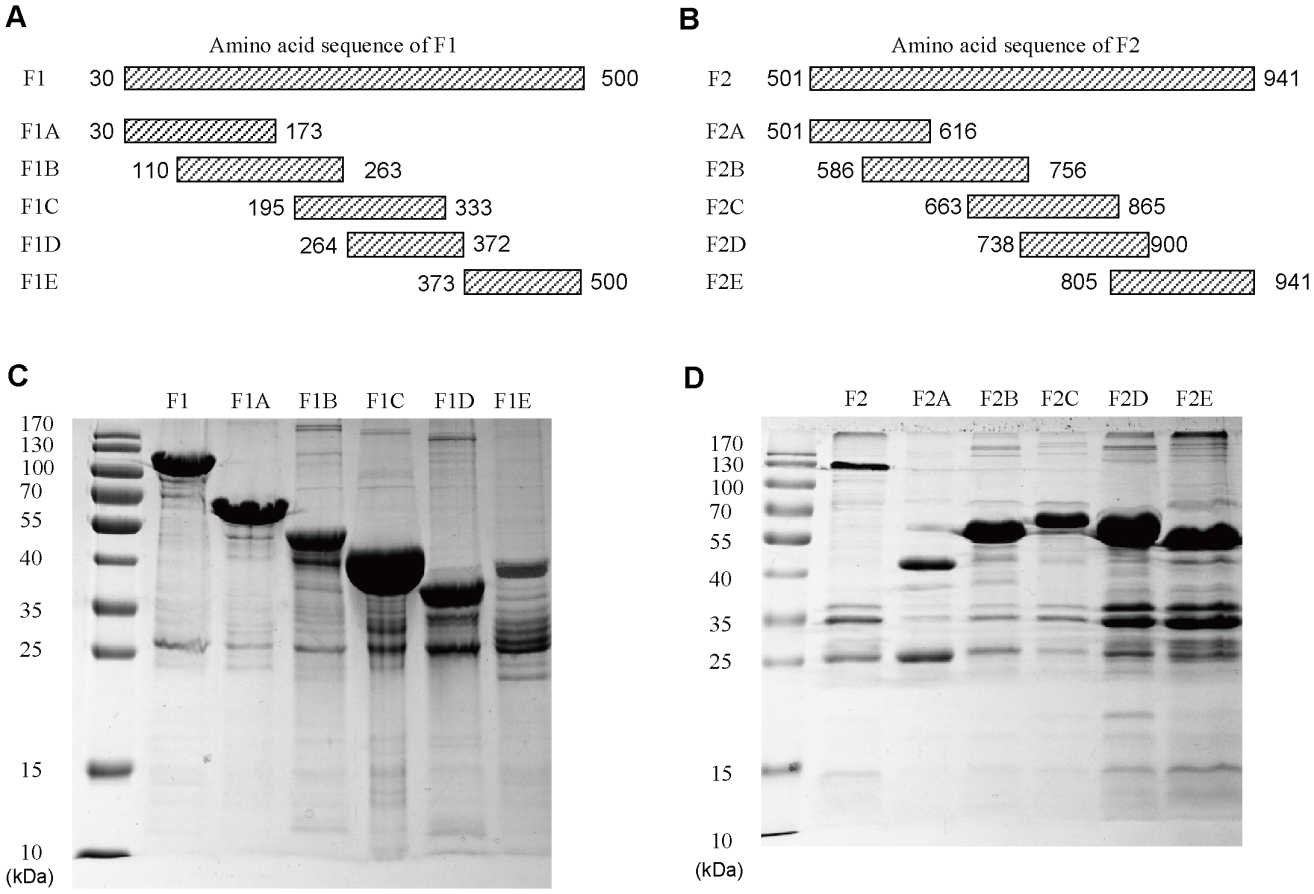
Generation of the fragments of F1 and F2. (A to B) A total of 12 different fragments were produced as GST fusion proteins from the fulllength FnBPA. (C to D) The quality of the GST fusion proteins, F1_30-500_, F1A_30-173_, F1B_110-263_, F1C_195-333_, F1D_264-372_, F1E_373-500_, F2_501-941_, F2A_501-616_, F2B_586-756_, F2C_663-865_, F2D_738-900_, and F2E_805-941_, was monitored in an SDS-polyacrylamide gel stained with coomassie blue dye. The molecular masses are shown on the left.

**Figure 5:**
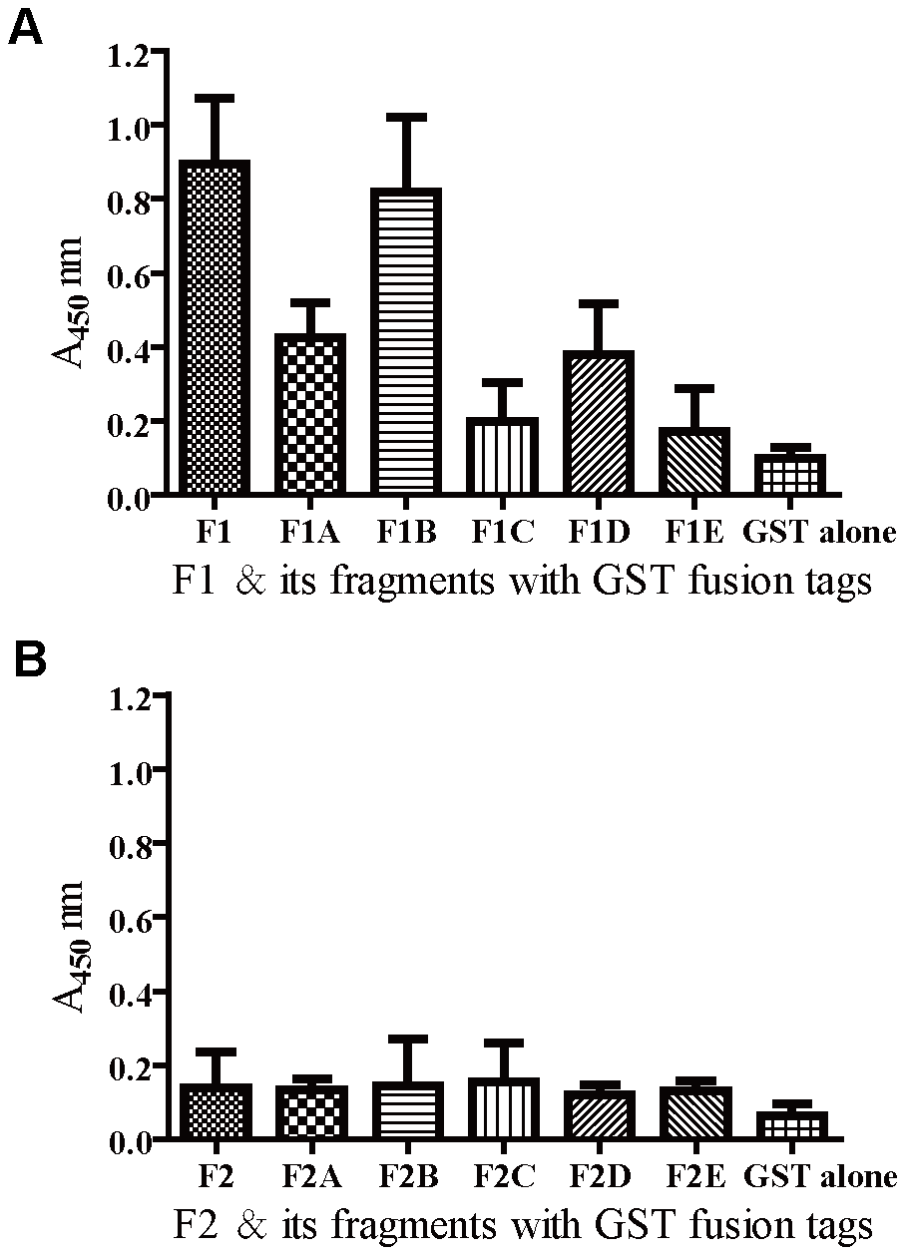
Reactivity of FnBPA fragments with mouse antisera. An ELISA plate was coated with the FnBPA fragments in the form of GST fusion proteins (displayed along the *x* axis) and reacted with each of the 20 mouse antisera at a dilution of 1∶1,000 (*y* axis). (A) The OD values obtained from the reactions of each fusion protein with the 20 mouse (immunization with F1 protein) antisera are expressed as means and standard deviations (*y* axis). (B) The OD values obtained from the reactions of each fusion protein with the 20 mouse (immunization with F2 protein) antisera are expressed as means and standard deviations (*y* axis).

To map the immunodominant regions of FnBPA, a total of 12 fragments (F1_30-500_, F1A_30-173_, F1B_110-263_, F1C_195-333_, F1D_264-372_, F1E_373-500_, F2_501-941_, F2A_501-616_, F2B_586-756_, F2C_663-865_, F2D_738-900_, and F2E_805-941_) were generated from FnBPA (Fig. 4A–B), and all were expressed as GST fusion proteins (Fig. 4C–D). These GST fusion polypeptides were reacted with each of the 20 mouse antisera. In detail, F1_30-500_, F1A_30-173_, F1B_110-263_, F1C_195-333_, F1D_264-372_, and F1E_373-500_ GST fusion proteins were reacted with each of the 20 mouse antisera from the mice immunized with F1 and alum. F2_501-941_, F2A_501-616_, F2B_586-756_, F2C_663-865_, F2D_738-900_, and F2E_805-941_ GST fusion proteins were reacted with each of the 20 mouse antisera from the mice immunized with F2 and alum. The OD values obtained from the reactions of F1 GST fusion protein with each of the 20 mouse antisera (immunization with F1 protein) was significantly higher than those from the the reactions of GST alone fusion protein (Fig. 5A). However, The OD values obtained from the reactions of F2 GST fusion protein with each of the 20 mouse antisera (immunization with F2 protein) were as low as those from the the reactions of GST alone fusion protein (Fig. 5B). Moreover, to identify the immunodominant regions of the truncated fragment F1, we compared the OD values obtained from F1A_30-173_, F1B_110-263_, F1C_195-333_, F1D_264-372_, and F1E_373-500_ GST fusion proteins with those from F1_30-500_. The results showed that the OD values obtained from the reactions of F1B_110-263_ GST fusion protein with each of the 20 mouse antisera (immunization with F1 protein) were as in a high level as those from the reactions of F1 GST fusion protein (Fig. 5A). These results suggest that the region covering residues 110 to 263 (F1B_110-263_) is highly immunogenic and is the immunodominant regions of FnBPA in mice immunized with the recombinant proteins.

### Immunization with the immunodominant regions of FnBPA (F1B_110-263_) generated protective immunity against systemic MRSA infection

**Figure 6:**
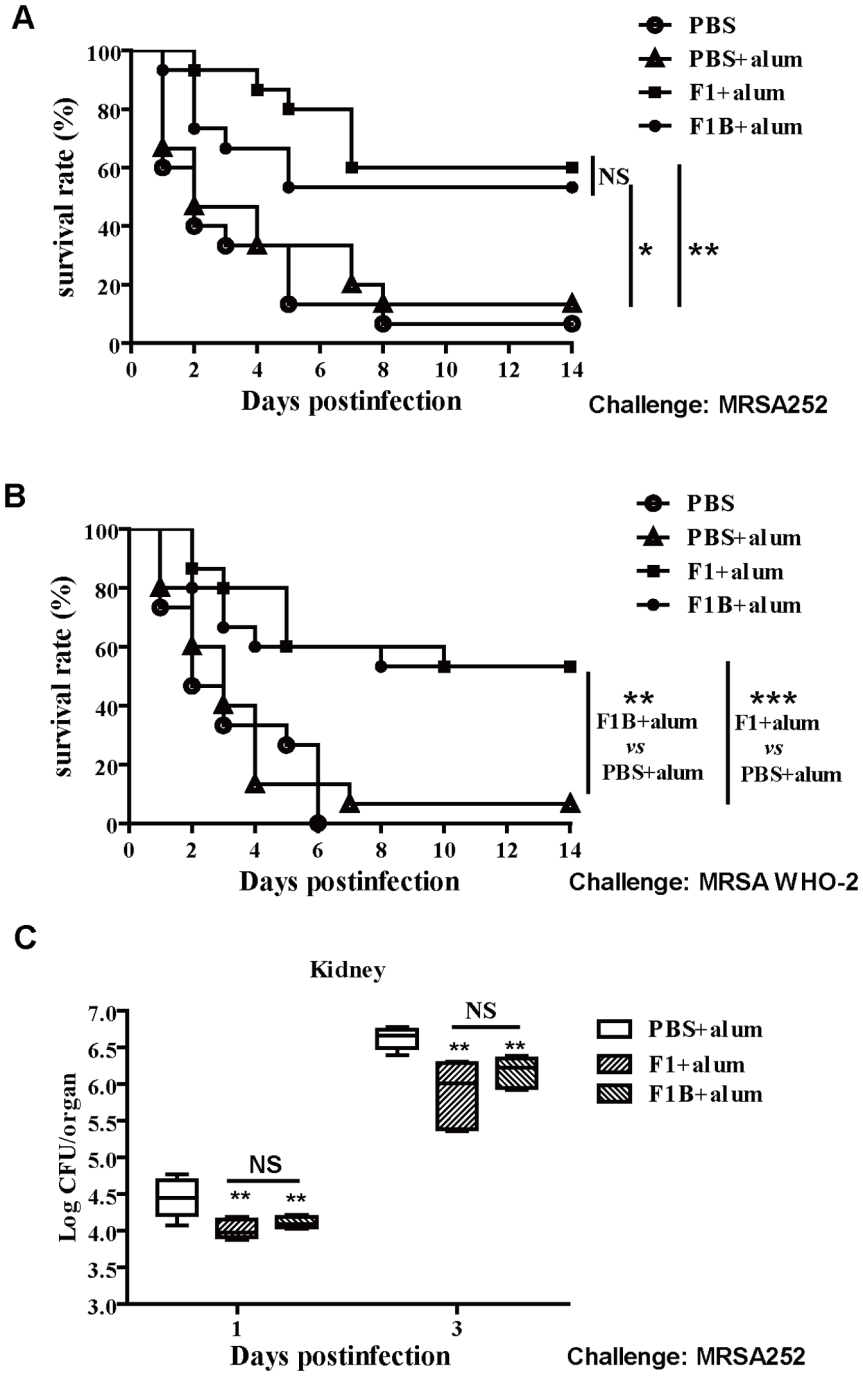
Immunization with the recombinant protein vaccine F1B_110-263_(F1B) generates protective immunity against systemic MRSA infection. BALB/c mice (n = 15) were immunized with individual antigens (F1, F1B) and alum adjuvant. The animals were challenged by intravenous injection of two different MRSA strains and were monitored for 14 days. Compared with animals receiving antigen-free PBS and the adjuvant alone, the significance of the protective immunity generated by the various antigens was measured with a log rank test. (A) *S. aureus* strain MRSA252 (challenge dose, 1×10^9^ CFU); (B) *S. aureus* strain WHO-2 (challenge dose, 1×10^9^ CFU). The asterisks represent a statistically significant difference (*P<0.05, **P<0.01, ***P<0.001). Representative results from one of three independent experiments are shown. (C)Bacterial numbers in kidneys of immunized and control mice were determined at 1and 3 days after infection with 2.5×10^8^ CFU i.v. Each group included 5 mice. Data are presented as box plots, and the medians and interquartile ranges are shown. Asterisks indicate significant differences between vaccinated and control mice (** *P*<0.01).

The mice were immunized with F1 or F1B_110-263_ three times at one- to two-week intervals. Fourteen days after the last immunization, the mice were infected via the tail vein with 1×10^9^ cells of different *S. aureus* strains (There are at least seven distinct isoforms of FnBPA which differ antigenically and exhibit limited immuno-crossreactivity [29]. MRSA252: isotype II; MRSA WHO-2: isotype III.). The mice vaccinated with the F1 or F1B_110-263_ antigen displayed higher survival rates (60%, 53.3% at 14 days respectively) than the alum adjuvant control group (13.3% survival) (challenge with MRSA252). The significance of protective immunity generated by the different antigens was measured with a log rank test (F1, *P* = 0.0021; F1B_110-263_, *P* = 0.0241.) (Fig. 6A). The mice vaccinated with the F1 or F1B_110-263_ antigen also displayed higher survival rates (53.3%, 53.3% at 14 days respectively) than the alum adjuvant control group (6.7% survival) (challenge with MRSA WHO-2). The significance of protective immunity generated by the different antigens was measured with a log rank test (F1, *P* = 0.0008; F1B_110-263_, *P* = 0.0055.) (Fig. 6B). These results showed that despite the low amino acid sequence similarity between the immunogen and the infecting strain, cross protection occurred. The results suggest that immunization with immunodominant regions of the FnBPA (F1B_110-263_) vaccine can generate partial protection against lethal challenge with two different *S. aureus* strains as well as that immunization with F1.

To determine whether the recombinant vaccine protects against bacterial growth *in vivo*, the kidneys from the immunized and control animals injected with the adjuvant alum were harvested and counted at days 1 and 3 after *S. aureus* MRSA252 infection (2.5×10^8^ CFU). The kidneys from mice actively immunized with the recombinant vaccine had lower levels of *S. aureus* than those in the control mice immunized with the alum adjuvant (Fig. 6C). These results suggest that the immune responses against the recombinant proteins were able to partially protect against *S. aureus* colonization. Intriguingly, in contrast to the immunization with F1, F1B_110-263_ vaccine, the immunodominant regions of the FnBPA, afforded a similarly high level of protection against *S. aureus* challenge.

### Opsonophagocytic killing activity of the antisera

**Figure 7:**
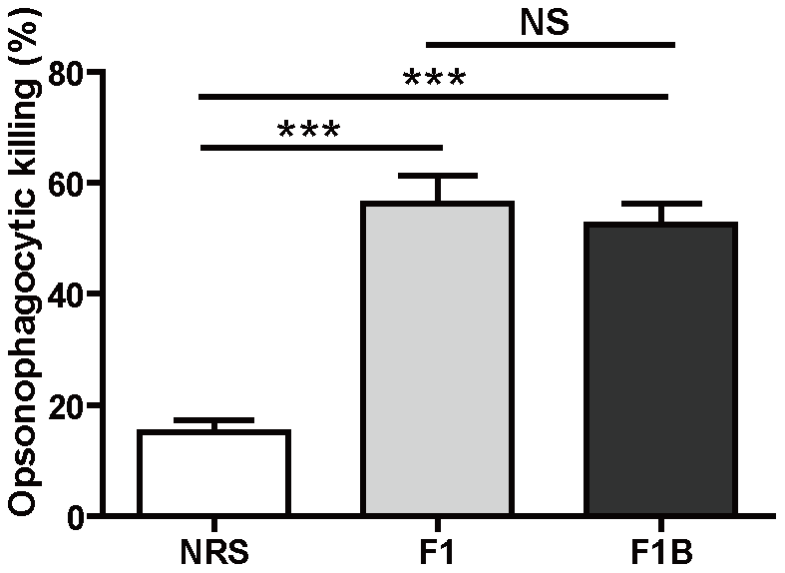
Opsonic activity of antibodies to FnBPA against the *S. aureus* MRSA252. *S. aureus* MRSA252 (1–2×10^5^ CFU per ml) was incubated in the presence of live leukocytes (1–2×10^6^ HL-60 cells per ml) and diluted rabbit antisera against F1, F1B_110-263_ or normal rabbit sera (NRS) in the presence of infant rabbit complement. They were plated on agar medium to measure bacterial survival as determined by CFU after 90 minute incubation. Then the percent of killing was calculated. The data shown are the means and the standard error of the means derived of 3 to 5 independent experiments. Unpaired 2 tailed student's t- tests were perfomed to analyze the statistical significance of data comparing non-reactive rabbit anti-serum with rabbit serum raised against specific antigens F1 (***P<0.001) and F1B_110-263_ (***P<0.001).

The opsonophagocytic killing by immune cells plays an important role in host clearance the *S. aureus*. To determine the nature of protection of antibodies against FnBPA, we analyzed their ability to induce opsonophagocytic killing of *S. aureus* in the presence of HL-60 cells and complement. HL-60 cells killing of *S. aureus* was monitored by using a bacterial burden assay. As shown in Figure 7, about 50% of *S. aureus* was killed by HL-60 cells when incubated with antibodies against F1 or F1B_110-263_ and infant rabbit serum with complement activity, and the percents of antibody mediated staphylococci killing significantly increased when serum was used from the rabbit immunized against F1 or F1B_110-263_ versus when antibodies were used from mock immunized rabbit. These results indicated the antibodies against FnBPA can induce opsonophagocytic killing of *S. aureus* in vitro.

## Discussion

*S. aureus* is a ubiquitous pathogen and also a major cause of nosocomial infections worldwide associated with high death rates, prolonged hospitalization and increased medical costs. Screening and defining *S. aureus* antigens will be the key to future vaccine development.

Fibronectin-binding proteins (FnBPs) A and B are members of the MSCRAMMs family of microbial proteins, which promote adhesion to tissue extracellular matrix, and are the most important adhesin molecules involved in the initial adhesion steps of S. aureus infection. Therefore, these molecules have been studied as potential vaccine candidates against *S. aureus* infection [30]. Previous studies independently confirmed the protective capacity of FnBPA in active vaccination and passive immunization. Specific systemic and mucosal immune responses can be elicited in mice using plasmid DNA-based vaccines encoding FnBP [31] and immunizations of rats with a truncated D2-domain of the Fnbp induced protection against endocarditis [25], and immunization other recombinant FnBPs also induced protective efficacy [32], [33].

In the present study, we defined the coordinates of FnBPA, from *S. aureus* strain MRSA252 based on the previous research that defined the coordinates of the A domain and the Fn binding repeats domains of FnBPA from *S. aureus* strain 8325 and based on the analysis of the amino acid sequence of the *S. aureus* strains [21], [29]. On the basis of the structural organization of FnBPA, we cloned and expressed the N-terminal and C-terminal of FnBPA (F1_30-500_ and F2_501-941_). We evaluated the immunogenicity of the two sections of FnBPA by ELISA and the protective efficacy of the two truncated fragments vaccines in a murine model of systemic *S. aureus* infection. The results showed recombinant truncated fragment F1 had a strong immunogenicity properties and the recombinant truncated fragment F2 had a poor antigenic properties. Survival rates significantly increased in the group of mice immunized with F1 than the control group. To futher identify the immunodominant regions of FnBPA, we cloned and expressed a total of 12 GST fusion proteins. The mouse antisera reactions suggest that the region covering residues 110 to 263 (F1B_110-263_) is highly immunogenic and is the immunodominant regions of FnBPA. Moreover, vaccination with F1B_110-263_ can generate partial protection against lethal challenge with *S. aureus* MRSA252 and reduced bacterial burdens against non-lethal challenge as well as that immunization with F1. We concluded that the F1B_110-263_ fragment was the immunodominant regions of FnBPA and can generate protective immunity against MRSA252 challenge.

In the previous studies, wann et al found the biological activity attributed to the N-terminal A regions of FnBPA [34]. The results showed that these regions exhibited substantial amino acid sequence identity to the A regions of other staphylococcal MSCRAMMs, including ClfA, ClfB, and SdrG, all of which bind fibrinogen. A recombinant form of the A region of FnBPA (rFnBPA_37-605_) can specifically recognize fibrinogen. Roche et al reported that the N-terminal A domain of FnBPA (rFnBPA_37-544_) promoted adhesion of *staphylococcus aureus* to elastin [35]. Keane et al found fibrinogen and elastin bound to the same region within the N-terminal A domain of FnBPA [21]. All these studies demonstrated that the ability of the N-terminal A domain of FnBPA to adhere to components of the extracellular matrix was an important mechanism for colonization of host tissues during infection. In our study, we found the N-terminal of FnBPA (F1_30-500_) had a strong strong immunogenicity and generated protective immunity. The protective mechanism is possible that the antibodies induced by immunization with F1 bind with the N-terminal A domain of FnBPA and inhibit the interaction between FnBPA and fibrinogen and elastin. Another mechanism is possible that vaccination induced the opsonophagocytic antibodies specific for *S. aureus* FnBPA, and this mechanism was confirmed by the opsonophagocytic killing analysis. However, according to the results of ELISA for specific antibodies, F2 subdomain, which is primarily composed of the fibronectin binding repeat region, is poorly immunogenic. The reason for low immunogenicity may be that the F2 region is intrinsically disordered [23], [36], [37]. Casolini et al reported the likelihood that the immunodominant epitopes were formed by the FnBP-Fn complex (ligand induced neo-epitopes), however, these antibodies are not protective [38]. The high affinity of FnBP for Fn ensures that as soon as the protein is in contact with serum a complex forms with Fn (tandem beta zipper). The dominant immune response to the Fn binding repeats is against the complex and therefore will not protect against infection.

To date, multiple attempts to develop a vaccine to prevent *S. aureus* infection have failed [39], [40], [41]. A single-antigen is not sufficient to achieve the goal of prevention of *S. aureus* infection. The inclusion of multiple staphylococcal antigens would likely result in a more effective vaccine. In our study, we further mapped the immunodominant regions of FnBPA and found F1B_110-263_ was highly immunogenic. Intriguingly, it generated protective immunity against MRSA252 challenge. This information will be important for further developing anti- *S. aureus* polyvalent subunit fusion vaccines.

In summary, the recombinant F1 improved the clinical outcomes in a murine model of systemic *S. aureus* infection by inducing humoral immunity. Moreover, the immunodominant regions of FnBPA have been identified. It achieved protective immunity against systemic *S. aureus* infection. However, further study is required to certify the biological activity of the antibodies elicited by vaccination in vitro, and prove that the protection is due to the immune response to FnBPA expressed on the surface of the infecting bacterium by testing a knockout mutant lacking the protein in the infection model and we will identify the epitopes of the immunodominant regions of FnBPA.
